# Bing-Neel Syndrome Case Report: A Previously Undocumented IgG Variant with MRI, PET/CT, and PET/MRI Imaging

**DOI:** 10.1155/2016/3931709

**Published:** 2016-04-06

**Authors:** Daniel Halperin, Simon Hallam, Athar Haroon, Tom Butler, Samir Agrawal

**Affiliations:** ^1^Whipps Cross Hospital, London E11 1NR, UK; ^2^St Bartholomew's Hospital, London EC1A 7BE, UK; ^3^Royal London Hospital, London E1 1BB, UK

## Abstract

Waldenstrom's macroglobulinaemia is the most commonly reported subtype of lymphoplasmacytic lymphoma (LPL); it is characterised by IgM secretion. Neurological complications are common usually as a result of hyperviscosity. In rare cases, cells can infiltrate the central nervous system; this is known as Bing-Neel syndrome. We report the case of a 57-year-old male with lymphoplasmacytic lymphoma of the IgG-subtype with neurological symptoms and the consequent finding of lymphoplasmacytoid cells in his cerebrospinal fluid as well as deposits on MRI and PET-CT imaging. This is the first report of Bing-Neel syndrome in IgG-subtype LPL. We discuss the biological and radiological markers of his disease, including PET imaging, which has been minimal in this area to date.

## 1. Introduction

Lymphoplasmacytic lymphoma is a small B-cell lymphoma defined by the WHO as being characterised by clonal B-cells with plasmacytoid differentiation and secretion of a monoclonal paraprotein, popularly referred to as Waldenstrom's macroglobulinaemia when the paraprotein is of the IgM class [1, 2]. As the majority of cases are IgM secreting, the terms LPL and WM are often used interchangeably. A small number of cases are IgG or IgA secreting and these are less well characterised in the literature.

Neurological complications occur in up to 25% of patients with LPL [3]. This most commonly presents as stroke or haemorrhage due to hyperviscosity or peripheral neuropathy from immunoglobulin deposition. Various pathophysiological mechanisms for neuropathy have been described in WM including limited evidence for antimyelin associated glycoprotein (anti-MAG) and direct infiltration of the nerves with IgM paraprotein [4]. However, there is no published data on non-IgM LPL.

Bing-Neel syndrome (BNS) is described as the presence of clonal plasmacytoid lymphocytes and plasma cells in the central nervous system (CNS) with or without cerebrospinal fluid (CSF) paraprotein and is an extremely rare manifestation of LPL [5]. Of the fifty or so case reports of BNS in the literature, all have been a complication of WM. We present the first recorded case of IgG secreting LPL causing BNS.

## 2. Case Report

A 57-year-old male presenting with fatigue and a microcytic anaemia was diagnosed in 2005 with lymphoplasmacytic lymphoma (LPL) with an immunoglobulin G (IgG) lambda serum paraprotein of 35 g/L.

In 2006, he received 3 cycles of single agent fludarabine without response but then achieved a partial response with combination of fludarabine-cyclophosphamide for a further 4 cycles. This was marked by a fall in his paraprotein from 35 g/L to 11 g/L.

He remained asymptomatic with minimal disease for the next two years. Bone marrow biopsy performed in January 2010 to investigate a drop in haemoglobin to 10.0 g/dL showed 9% involvement of LPL in the marrow. At this time his serum IgG concentration was 20 g/L and his B2 microglobulin was raised at 5.3. By May 2010 his Hb had dropped to 9.1 and he was feeling progressively more fatigued. CT-PET showed increased uptake in the cervical, axillary, and external iliac lymph node chains as well as mild splenomegaly. There was no significant change on the bone marrow biopsy from the previous result.

He received 6 cycles of fludarabine, cyclophosphamide, and rituximab in June 2010 which achieved a complete metabolic response on CT-PET and resolution of the anaemia (14.1 g/dL).

He then presented in June 2014 with a 2-month history of transient visual disturbance, ataxia, and gradual, subtle cognitive decline. He had been having headaches for 11 months which had been attributed to migraines. He had also lost 6 kg in the preceding 3 months.

On examination there was no palpable lymphadenopathy or hepatosplenomegaly. The full blood count was normal and bone marrow trephine biopsy showed less than 10% infiltration with B-cell lymphoma. PET/MRI showed metabolically active soft tissue in the paravertebral region of L4-L5 with abnormal epidural tissue in L3/L4 and L5/S1 level. There was moderate metabolic activity at L4 vertebral body.

**Figure 1:**
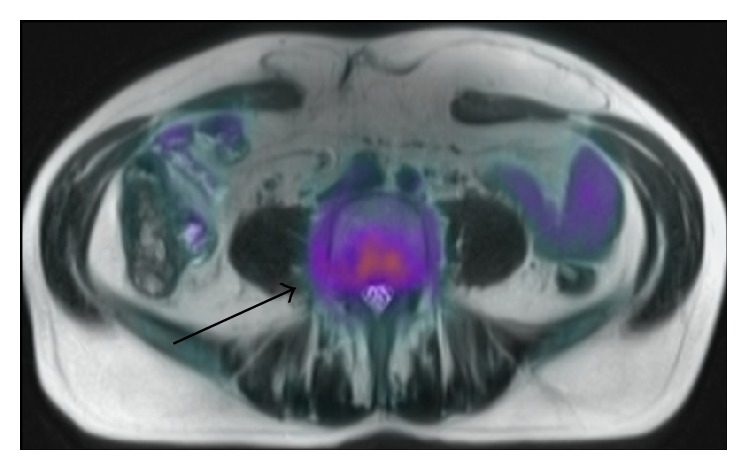
CT-PET axial segment showing high uptake in the paravertebral soft tissue regions at L4/L5.

**Figure 2:**
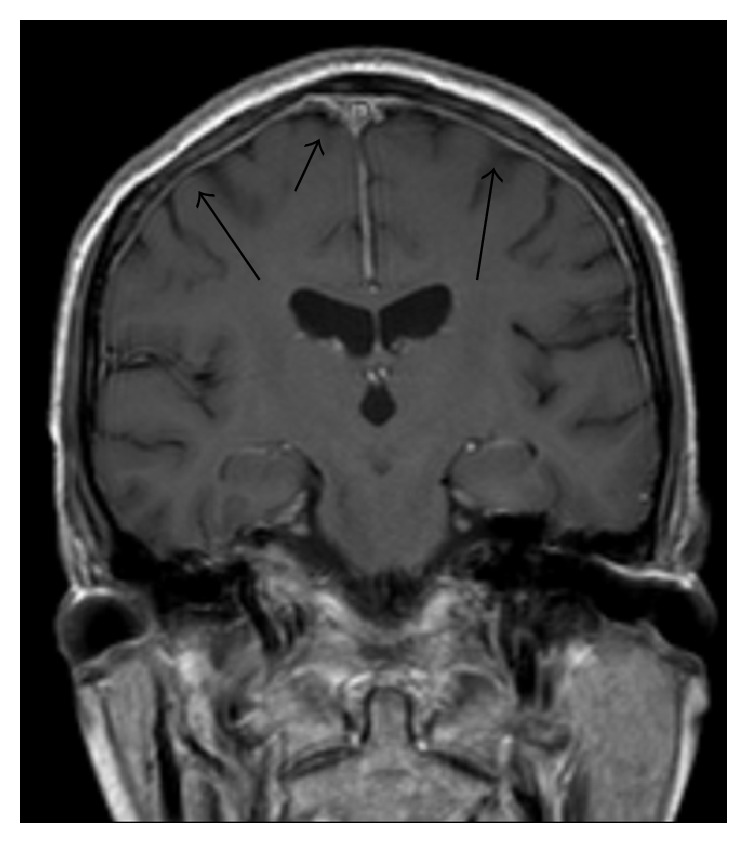
MRI brain coronal view shows diffuse meningeal enhancement.

**Figure 3:**
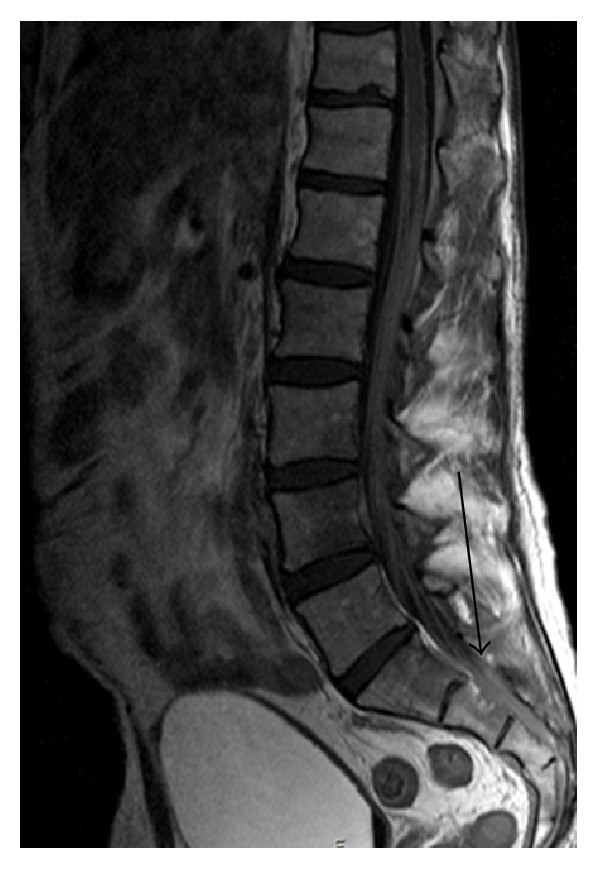
MRI lower thoracic and lumbar spine sagittal view showing enhancement of the distal spinal cord and conus medullaris.

PET MRI axial fused image [Figure 1], diffuse meningeal enhancement on coronal MRI brain [Figure 2], and a sagittal MRI spine demonstrated enhancement of the distal cord and conus medullaris [Figure 3].

**Figure 4:**
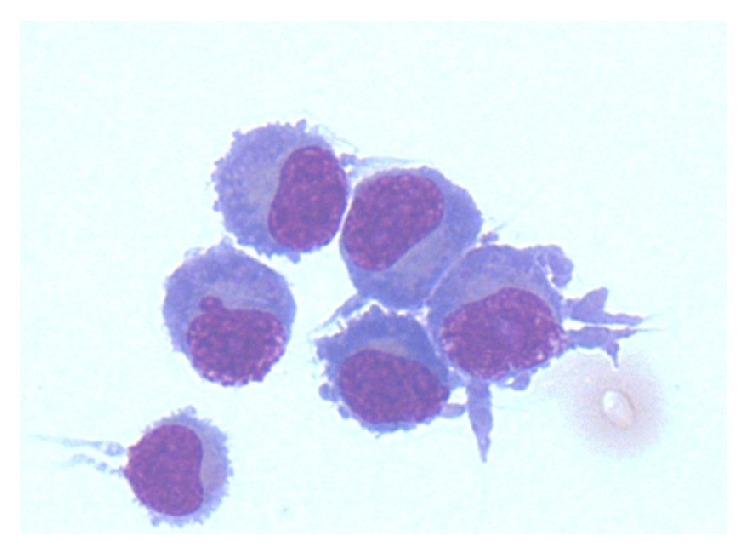
Plasmacytoid cells found on cytospin of the cerebrospinal fluid confirming cellular infiltration of the central nervous system.

Cerebrospinal fluid (CSF) analysis revealed elevated oligoclonal IgG and a white cell count of 98 cells per microlitre. The oligoclonal bands consisted of one prominent beta 2/fast gamma band and one smaller mid/slow gamma zone band suggesting a smaller separate clonal population. CSF cultures grew no bacterial or fungal organisms and viral PCR was negative. A cytospin preparation of CSF revealed numerous plasmacytoid cells with some plasma cells and small lymphocytes [Figure 4]. CSF immunophenotyping identified a CD5-negative CD23-positive lambda-restricted B-cell clone, also found in the bone marrow. The IgG paraprotein level was 1,220 g/L. A diagnosis of LPL stage IV-B with low level bone marrow involvement and CNS infiltration was made. Importantly there were no other sites of disease and no evidence of transformation to high grade lymphoma.

CNS-directed therapy was instituted with high-dose intravenous methotrexate; however, after three cycles a lack of response was documented with unchanged meningeal enhancement on imaging and presence of clonal B-cells in the CSF. Therefore, treatment was changed to high-dose cytarabine and a complete remission was achieved after two cycles. In view of the extensive past treatment, plus the difficulty in clearing the CNS disease, it was considered that an autologous peripheral blood stem cell transplant (PBSCT) was warranted to consolidate the response, although PBSCT is rarely used for LPL. The PBSCT was performed using BCNU and Thiotepa chemotherapy for conditioning a regimen used for primary CNS lymphoma because of its CNS activity.

After transplant there has been mild improvement in the enhancement pattern in the cord with new areas of very localised signal abnormality in the subcortical white matter of the right posterior temporal lobe and right precentral gyrus at the vertex without enhancement. These new areas were thought to be treatment related.

He has had thorough investigation for hyponatraemia since February 2015, presenting to Accident and Emergency with headaches, confusion, and a serum sodium concentration of 123 mmol/L. He was treated with low dose hydrocortisone for low cortisol levels and currently has no neurological symptoms. Six months after transplant he is clinically well and on imaging there is still some abnormal enhancement over the roots of the cauda equina with patchy but stable signal on MRI at L4 and S1 indicating low level disease.

## 3. Discussion

In 1936 Danish physicians Bing and Neel described a triad of hyperglobulinaemia, neurological symptoms, and presence of lymphocytes within the central nervous symptoms [5]. Remarkably this was 8 years before Waldenstrom's macroglobulinaemia was defined as a clinical entity [2].

Numerous attempts have been made to categorise Bing-Neel syndrome. The most commonly referred to classification is of “tumoural” and “diffuse” subtypes [6]. Tumoural type is characterised by focal neurological deficits and seizures whilst diffuse cases present with headaches, behavioural change, and decline in cognition. Our patient's headaches and slow neurological decline arguably fall under the diffuse category.

The challenge for the acute physician or general haematologist is to distinguish symptoms caused by hyperviscosity from direct cellular infiltration. In WM, the IgM paraprotein level in the blood is closely related to the risks of hyperviscosity syndrome [7]; however, there is no relationship between IgM levels and the risk of BNS. In non-IgM forms of LPL, the risk of hyperviscosity is considerably lower and the occurrence of neurological symptoms should evoke consideration of BNS.

The most commonly reported symptoms of BNS are headache, ataxia, and progressive cognitive decline [8]. Focal neurology of the tumoural subtype includes ptosis, facial droop, and dysarthria.

Blood tests usually show the typical features of LPL including a normocytic normochromic anaemia, elevated ESR often >100 mm/hour, monoclonal gammopathy on serum electrophoresis, and occasionally presence of Bence-Jones protein; however, there are no specific features to suggest BNS.

Diagnosis is made on cerebrospinal fluid testing and radiological imaging. Typical CSF findings include an elevated opening pressure, presence of lymphocytes between 100 and 500 mm, and a monoclonal band, although with the IgM cases there has typically been no link to the amount of protein in the CSF and presence of BNS or its severity [9]. Cytology and immunophenotyping of CSF are key in making a diagnosis: lymphoplasmacytoid cells and mature plasma cells morphologically, with clonal B-cells immunologically (typically, light chain restricted, CD5-negative B-cells) [10]. MDY88 mutations have a role in differentiating LPL from other B-cell neoplasms with plasmacytic features but have no association with neurological infiltration [11]. Our patient was diagnosed in 2005 before MDY88 became routine in clinical practice.

Whilst CT scanning can detect larger lymphoma nodules in the tumour form of the disease, most findings are found on MRI T1 weighted postgadolinium imaging as leptomeningeal enhancement in the diffuse form [7, 12]. To our knowledge, this is the first case described in the literature outlining the findings on PET/CT and PET/MRI. Metabolic imaging demonstrates extent of disease by showing metabolically active foci at involved sites and compliments the role of routine contrast enhanced MRI of the brain by detecting extracerebral sites of disease.

There are no published guidelines on management of this condition although a number of regimes have been used. The most recurrent successful outcomes are associated with the use of intrathecal methotrexate and purine analogues [6, 9, 13, 14]. Ibrutinib has also been used with success especially on patients with MYD88 mutations. Little data exists on its use in either non- IgM LPL or BNS [15]; however, there has been a recent case report describing successful use of ibrutinib in IgG LPL suggesting potential for future use in this context [16].

In our patient, the widespread FDG-avid lesions throughout the CNS and his presentation suggested that the LPL was behaving more like an aggressive lymphoma. Given his age and good performance status, plus his extensive prior purine analogue (fludarabine) therapy, he was treated with CNS-directed high-dose chemotherapy and consolidation with auto-PBSCT. The use of a primary central nervous system lymphoma conditioning regime pretransplant has not been reported before. Its long term success remains to be seen.

## Additional Points

This is a unique case to our knowledge and could be classified as a new entity or a new variant of an existing rare syndrome.
